# Analyses of the Transcriptome and Metabolome Demonstrate That HIF1α Mediates Altered Tumor Metabolism in Clear Cell Renal Cell Carcinoma

**DOI:** 10.1371/journal.pone.0120649

**Published:** 2015-04-01

**Authors:** Denise R. Minton, Leiping Fu, Qiuying Chen, Brian D. Robinson, Steven S. Gross, David M. Nanus, Lorraine J. Gudas

**Affiliations:** 1 Department of Pharmacology, Weill Cornell Graduate School of Medical Sciences, New York, New York, United States of America; 2 Department of Pathology, Weill Cornell Medical College, New York, New York, United States of America; 3 Division of Hematology and Medical Oncology, Department of Medicine, Weill Cornell Medical College, New York, New York, United States of America; 4 Weill Cornell Meyer Cancer Center, Weill Cornell Medical College, New York, New York, United States of America; Baylor College of Medicine, UNITED STATES

## Abstract

Hypoxia inducible factor 1 alpha (HIF1α) is a transcription factor that is frequently stabilized and active in human clear cell renal cell carcinoma (ccRCC). We have found that constitutively active HIF1α is sufficient to cause neoplastic transformation in a murine model of ccRCC termed the TRACK model. RNA sequencing (RNAseq) and untargeted metabolomics analyses of samples from TRACK kidneys demonstrate that HIF1α activates the transcription of genes that cause increased glucose uptake, glycolysis, and lactate production, as well as a decrease in the flux of pyruvate entering the tricarboxylic acid (TCA) cycle and a decrease in oxidative phosphorylation; these changes are identical to those observed in human ccRCC samples. These studies show that a constitutively active HIF1α promotes tumorigenesis in TRACK mice by mediating a metabolic switch to aerobic glycolysis, i.e., the *Warburg effect*, and suggest that TRACK mice are a valid model to test novel therapies targeting metabolic changes to inhibit human ccRCC.

## Introduction

Each year, kidney cancer affects nearly 300,000 people and causes close to 100,000 deaths worldwide [1]. The majority of kidney cancer cases are clear cell renal cell carcinomas (ccRCCs), which are commonly linked to inactivating mutations of the von Hippel-Lindau tumor suppressor gene (*VHL*) [2]. The protein encoded by this gene, pVHL, regulates the activation of a family of transcription factors, hypoxia inducible factors (HIFs), that mediate the primary transcriptional responses to hypoxia in normal and neoplastic cells [3]. In ccRCC, pVHL inactivation results in aberrant regulation of HIF1α; this transcription factor accumulates, translocates to the nucleus, and binds to HIF1β [3,4]. This HIF1α/HIF1β dimer then binds to hypoxia response elements (HREs) on DNA to activate the transcription of target genes that are involved in angiogenesis, glucose uptake, enhanced glycolytic metabolism, and other processes related to cell proliferation [3,4].

While it is largely accepted that HIF1α is the more tumorigenic isoform in most types of cancer [3], some researchers have suggested that HIF2α is more tumorigenic than HIF1α in ccRCC [5]. One piece of data consistent with this idea is that HIF2α knockdown prevents the growth of tumors in a xenograft model, while HIF1α knockdown does not [6–8]. However, these experiments do not assay for tumor development but rather for proliferation of tumor cells. An additional piece of data is that a loss of a portion of chromosome 14q near the location of *HIF1α* occurs frequently in ccRCC [9]. However, RNAseq data from The Cancer Genome Atlas Research Network show that *HIF1α* mRNA remains at a level within the range of that in tumors that are diploid for HIF1α in most ccRCC specimens that are affected by *HIF1α* loss of heterozygosity [10]. Consistent with these data, several groups have shown that HIF1α is highly expressed in a large number of ccRCC cases [11–13], and that high HIF1α levels in tumors are associated with significantly lower patient survival [14]. Most recently, one group demonstrated expression of HIF1α by immunostaining in 84% of 106 primary surgical ccRCC specimens [15].

We previously reported that expression of a constitutively active form of HIF1α can drive tumorigenesis in a murine model of ccRCC called the TRACK (**TRA**nsgenic model of **C**ancer of the **K**idney) model [16]. TRACK mice express a mutated, constitutively active HIF1α that drives a program of tumorigenesis specifically in renal proximal tubules, and this tumorigenesis program mimics many features of early human ccRCC both phenotypically and at the molecular level [16]. TRACK kidney histologies display areas of distorted tubular structures, cells with clear cytoplasm and increased glycogen and lipid deposition, multiple renal cysts, and early onset of ccRCC [16]. These mice, however, do not develop metastases and the mice do not die prematurely. In contrast, we and others have shown that expression of a mutated, constitutively active HIF2α in the proximal tubules of mice does not result in neoplastic transformation and ccRCC [17,18]. The transgenic mice our lab generated that express constitutively active HIF2α in the proximal tubule cells develop glycogen deposits, but no tumors [17]. Collectively, these findings indicate that HIF1α plays a critical role in promoting renal tumorigenesis.

Altered tumor metabolism is now a widely accepted hallmark of cancer. Metabolic alterations accompany tumorigenesis and can be causal for tumor development and progression [19]. HIF1α is largely responsible for activating the transcription of target genes that drive various features of aberrant tumor metabolism, such as increased glucose uptake, increased glycolysis and lactate production, and decreased mitochondrial respiration [20,21]. Collectively, these features of tumor metabolism are commonly referred to as the *Warburg effect* [22]. The Warburg effect is a process in which cells rely on aerobic glycolysis instead of mitochondrial oxidative phosphorylation to generate energy, even though glycolysis is a less efficient pathway for producing ATP. Despite the relative bioenergetic cost of aerobic glycolysis, this shift in metabolism can confer an advantage by facilitating the generation of biomass needed to produce a new cell, and thus cancer cells acquire and metabolize nutrients in a manner that is conducive to proliferation rather than efficient ATP production [22].

Here we report that kidneys from TRACK mice exhibit increased expression of HIF1α target genes that have been linked to a shift in metabolism from mitochondrial oxidative phosphorylation to an accelerated rate of aerobic glycolysis and lactate production, similar to what is observed in human ccRCC. Additionally, we report metabolomics data and show that both the TRACK kidneys and human ccRCC samples exhibit increases in glycolytic intermediates and lactate, in association with a decrease in metabolites of the TCA cycle. Together, these data implicate HIF1α in mediating alterations in kidney metabolism that drive tumorigenesis, and suggest that TRACK mice represent a valid model to test therapies that target metabolism with the goal of inhibiting ccRCC.

## Materials and Methods

### Samples

Male C57BL/6 mice (Jackson Laboratories) and TRACK mice were generated as described [16]. Three TRACK (TG^**+**^) and three wild-type littermates (TG^**−**^) males, 18 months old, were selected for RNAseq analysis. Five TG^**+**^ and five TG^**−**^ mice, 12 months old, were selected for metabolomics analyses. Older mice were selected for these experiments because they display a more advanced disease, which we hypothesized would more closely mimic features of human ccRCC. All procedures involving the use of mice were approved by the Weill Cornell Medical College (WCMC) Institutional Animal Use and Care Committee.

The WCMC Institutional Review Board (IRB) approved the use of human tissue samples for this study. A consent form is signed by all patients undergoing surgery, which states the tissue may be used for research purposes if tissue is collected and de-identified; the IRB approved this exemption/expedited protocol. ccRCC and adjacent normal kidney tissue samples were obtained from 12 patients who underwent radical or partial nephrectomy at WCMC. These samples were embedded in Optimal Cutting Medium (OCT), snap frozen, and stored at −80°C. Hematoxylin and Eosin slides were prepared of the frozen tissue samples to ensure that the areas selected for metabolomics analysis represented either pure ccRCC or normal kidney tissue.

### RNA Sequencing

High quality RNA was extracted from a thin layer of kidney cortex tissue (n = 6) using mini-RNAeasy columns (Qiagen) and RNAs were converted to cDNA by semi-quantitative reverse transcriptase (RT)-PCR. The WCMC Genomics Core performed the sequencing (51-bp single-end reads) on an Illumina HiSeq2000 Sequencer and data were analyzed as previously described [17]. In brief, data analysis was mainly performed with the Tuxedo tools software [23]. A stringent threshold was used to select differentially expressed genes (fold change >2 or <0.5, q<0.01). 655 genes showed increased mRNA levels and 55 genes showed decreased mRNA levels in TRACK TG+ kidney samples compared with the TG− control kidney cortex samples. Data are in the GEO database (accession no. GSE54390).

### Oncomine

The Oncomine Cancer Microarray database (http://www.oncomine.org) was used to study mRNA levels of genes most highly expressed in human ccRCC tumor types versus normal kidney tissue [24]. The data collected were from five different data sets [25–28], each of which included data from 20–260 patient samples. For our analysis, we searched for the genes most highly overexpressed in human ccRCC versus normal healthy renal tissue. Oncomine sorts overexpressed genes by “median rank,” which is the median p-value rank across data sets. For our purposes, we analyzed the top 30 ranked genes (S1 Table).

### Metabolite Extraction

Mouse kidneys were isolated and snap-frozen in liquid nitrogen. Human tissue was embedded in OCT medium, snap frozen, and stored at −80°C until the day of metabolite extraction. Tissue samples were washed twice with cold PBS, followed by bead-beating in 80% −70°C methanol:water (LC-MS grade methanol, Fisher Scientific) using a Tissuelyser cell disrupter (Qiagen). The extraction mixture was incubated at 4°C for 10 min, and then centrifuged for 5 min at 13.2k rpm to separate the protein pellet. The extraction procedure was repeated twice. The supernatants were pooled, dried in a speed-vac (Savant) and stored at −80°C. The pellets were solubilized in 200 μl of 0.2M NaOH by incubating at 95°C for 20 min, and protein contents were quantified with BioRad DC protein assays. Kidney metabolites were normalized to protein for LC/MS metabolomic analysis.

### LC-MS and LC-MS/MS Platforms for Metabolite Profiling

The LC/MS and LC MS/MS instrumentation and setup were the same as described [29], except for the LC mobile phase composition and gradient. For the aqueous normal phase (ANP) separation, mobile phase A consisted of 0.025% acetic acid and 6 μM EDTA in 50% isopropanol: 50% ddH_2_O; mobile phase B consisted of 5 mM ammonium acetate and 6 μM EDTA in 90% acetonitrile (ACN). Gradient steps were: 0–1 min, 99% B; 1–15 min, to 0% B; 15–29 min, 0% B; 29–37 min, 99% B. The LC/MS data were acquired in both positive and negative ionization modes.

### Metabolomics Data Processing and Statistical Analysis

Raw data files were processed by Agilent MassHunter Qualitative Analysis Software and analyzed by statistical analysis in Mass Profiler Professional (Agilent Technology, MPP, version B2.02) [29]. Aligned molecular features detected in all biological replicates of at least one group were directly applied for statistical analysis across treatment groups by MPP. The Benjamini Hochberg FDR correction was applied for multiple testing correction of p-values in one-way ANOVA (corrected p<0.05). An uncorrected p-value was used when individual metabolites were manually inspected for statistical significance between two groups (e.g. simple student t-test). Differential metabolites were identified using the METLIN Personal Metabolite Database (Agilent Technologies) and a molecular formula generator (MFG) algorithm in MPP as previously explained in detail [30].

## Results

### Analysis of mRNA Expression in TRACK Kidneys and Human ccRCC Samples

We have previously shown that expression of a constitutively active, mutant form of HIF1α in the proximal tubules causes tumorigenesis in the TRACK mouse, a murine model of ccRCC [16]. These observations prompted us to delineate the genes that HIF1α activates in the TRACK mice. We performed RNAseq analyses on TRACK vs. wild type, transgenic negative (TG^**−**^) kidney cortex samples and compared the results to mRNA expression data from Oncomine, a microarray database that compares gene expression in human cancers vs. normal tissue [24]. Specifically, we used this database to analyze results from five data sets of ccRCC, each of which included data from 20–260 patients samples [25–28]. For the purpose of this study, we focused on the top 30 overexpressed genes (ranked by median p-value rank across datasets) (S1 Table).

Many of the genes overexpressed in both human ccRCC and TRACK kidneys are implicated in altered tumor metabolism (Table 1). Carbonic Anhydrase IX (CAIX, Gene ID: 768; mRNA increased 10-fold in TRACK mice and 16-fold in ccRCC) is a HIF1α target gene and a major biomarker of ccRCC [31,32]. It encodes a zinc metalloprotein anchored to the extracellular side of the plasma membrane and is believed to regulate extracellular pH for support of anaerobic tumor metabolism [33]. NADH dehydrogenase 1 alpha subcomplex, 4-like 2 (NDUFA4L2, Gene ID: 56901; mRNA increased >50-fold in both TRACK kidneys and ccRCC) is a subunit of complex 1 in the electron transport chain; upregulated by HIF1α, it inhibits oxidative phosphorylation and thus promotes the shift in metabolism to glycolysis [34]. Phosphofructokinase, platelet (PFKP, Gene ID: 5214; mRNA increased 10-fold in TRACK mice and 7-fold in ccRCC) encodes a kinase enzyme that catalyzes the conversion of fructose-6-phosphate to fructose-1,6-bisphosphate during the third step of glycolysis. This irreversible step in glycolysis is an important control point, and in tumors, PFKP plays a role in accelerating the rate of glycolysis [35,36]. Pyruvate dehydrogenase kinase, isoenzyme 1 (PDK1, Gene ID: 5163; mRNA increased 7.5-fold in TRACK kidneys and nearly 5-fold in ccRCC) phosphorylates and inactivates pyruvate dehydrogenase, an enzyme that converts pyruvate to acetyl-coenzyme A. Thus, PDK1 inhibits pyruvate metabolism via the TCA cycle and in turn inhibits oxidative phosphorylation [37]. Solute carrier family 2 (facilitated glucose transporter), member 1 (SLC2A1 or GLUT1, Gene ID 6513; mRNA increased 5-fold in TRACK kidneys and 2.5-fold in ccRCC) is not one of the top 30 ranked genes in human ccRCC according to Oncomine data, but SLC2A1 has been well studied in ccRCC and is known to play an important role in altered metabolism. More specifically, SLC2A1 is upregulated in cancers to accelerate the uptake of glucose and meet the energy needs of the tumor for an accelerated rate of glycolysis [21]. Lastly, solute carrier family 16, member 3 (SLC16A3 or MCT4, Gene ID: 9123; mRNA increased 87-fold in TRACK mice and nearly 7-fold in ccRCC) is a monocarboxylate transporter that is upregulated in many tumors because it is required for lactate secretion out of the cell, pH homeostasis, and maintenance of the Warburg effect [38]. With the exception of SLC2A1, which is a target gene of both a HIF1α and HIF2α, the other genes discussed are only regulated by HIF1α [21]. Thus, HIF1α is implicated in activating the transcription of genes associated with aerobic glycolysis in both the TRACK model of ccRCC and human ccRCC.

**Table 1:** Comparison of TRACK kidney gene transcripts with human clear cell renal cell carcinoma.

Gene	Description	Fold Increase in ccRCC	Fold Increase in TRACK
CAIX	Carbonic Anhydrase IX	16.2	9.8
NDUFA4L2	NADH dehydrogenase 1 alpha subcomplex, 4-like 2	52.0	60.3
PDK1	Pyruvate dehydrogenase kinase, isoenzyme 1	7.0	7.5
PFKP	Phosphofructokinase, platelet	7.2	9.8
SLC2A1	Solute carrier family 2 (facilitated glucose transporter), member 1	2.5	4.7
SLC16A3	Solute carrier family 16, member 3	6.9	86.5

Selected genes that are most highly expressed in human ccRCC, and which show increased mRNA levels (fold change > 2) in the kidney tissue of the TRACK mice relative to kidneys from wild type littermates. Human data were collected from Oncomine, and TRACK data were obtained by RNAseq.

### Untargeted Tissue Metabolite Profiling

We next performed metabolomic analyses on TRACK kidney samples to determine if the increases in mRNA levels observed for HIF1α target genes implicated in altered tumor metabolism are, in fact, associated with changes in metabolite levels in the TRACK kidneys. For comparison, we also extracted metabolites from primary human ccRCCs, along with adjacent normal kidney tissue as control. Principal component analysis (PCA) plots of the metabolomics data collected by both hydrophilic, aqueous normal phase chromatography with negative ion mode mass spectrometry (MS) detection (ANP-NEG) and positive ion mode MS detection (ANP-POS) show that the TG^**−**^ and TRACK samples are clearly distinguished, as are normal human kidney and ccRCC samples, which reflects distinct differences in the respective metabolomes (S1 Fig. A,B). Consistent with the unsupervised pattern recognition found by PCA, the hierarchical cluster analysis (HCA) dendogram similarly shows reproducible patterns of within-group similarity and between-group differences in the metabolites for each of the four groups analyzed (S1 Fig. C,D). Notably, for the human samples relative to mouse, tumors are genetically heterogeneous, explaining the greater metabolite variability for within-group similarities and between-group differences.

### Analysis of Metabolic Changes in TRACK Kidneys

**Fig 1:**
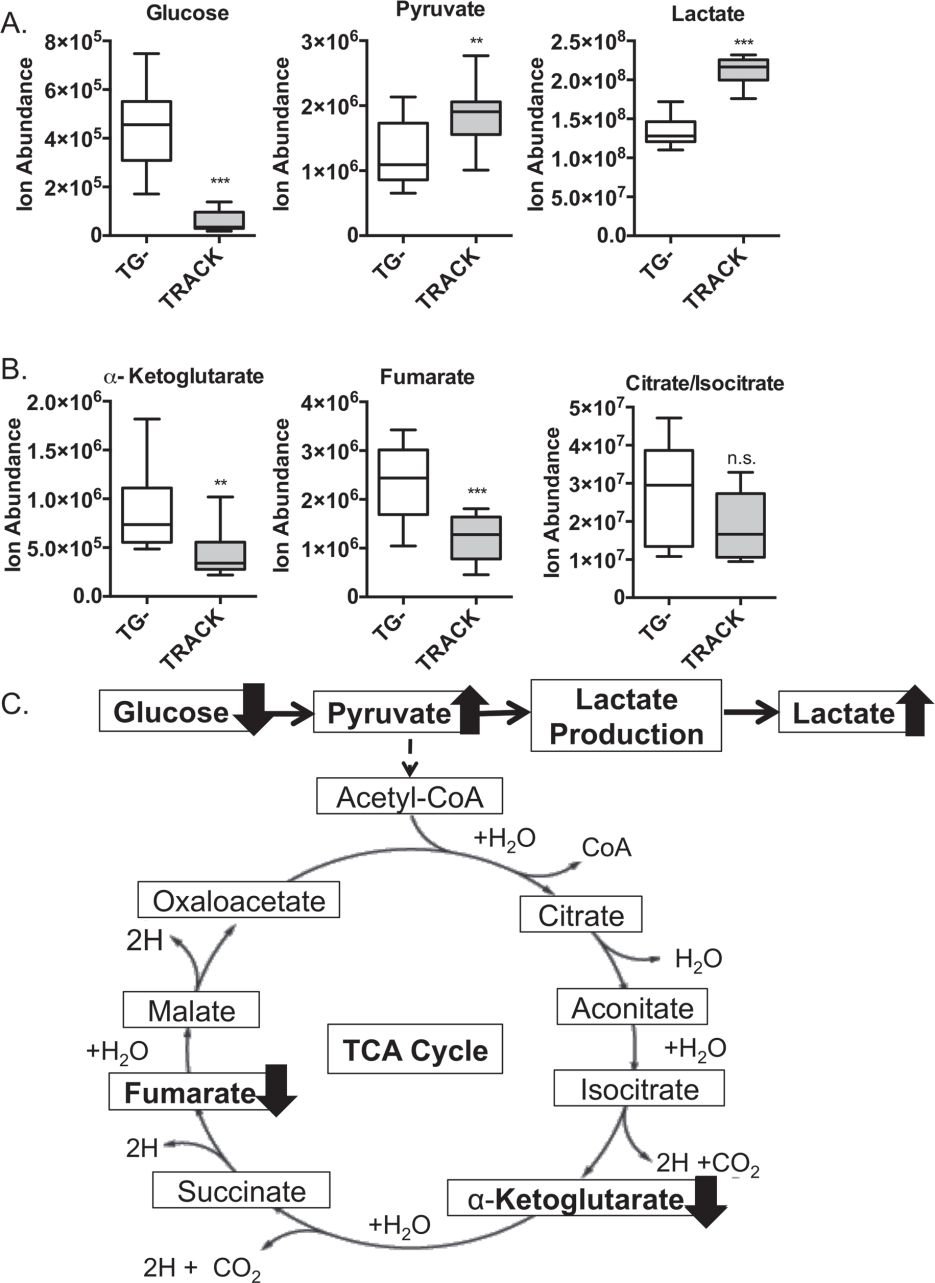
Metabolomic analysis of key intermediates in glycolysis, lactate production, and the TCA cycle in TRACK kidneys. A: Comparison of the levels of glycolysis and lactate production intermediates, glucose, pyruvate, and lactate in TG^**−**^ vs. TRACK kidney samples (n = 14 and 12). □ = TG^**−**^; ■ = TRACK. B: Comparison of the levels of fumarate, α-ketoglutarate, and citrate/isocitrate in WT vs. TRACK kidney samples (n = 14 and 12). □ = TG^**−**^; ■ = TRACK. C: Schematic overview of changes to glycolysis, lactate production, and the TCA cycle in TRACK kidneys. Error bars indicate the mean ± SD. * indicates a p value <0.05, ** indicates a p value <0.01, and *** indicates a p value <0.001.

Prior studies have shown that in ccRCC, HIF1α upregulates expression of genes that mediate glucose uptake, SLC2A1 and SLC2A3, along with several glycolytic enzymes, including HK2, PFK-1, and LDHA, in accord with an oncogenic switch to aerobic glycolysis [3,21]. We therefore performed targeted inspection of the metabolite profiling data to assess whether lactate and glycolytic intermediates accumulate in TRACK kidneys. As illustrated in Fig. 1A, glucose levels are diminished, while pyruvate and lactate levels are significantly elevated, suggesting that the glycolytic and lactate production pathways are upregulated in TRACK kidneys (Fig. 1C).

Under (aerobic) conditions in most tissues pyruvate efficiently enters the TCA cycle and drives production of NADH, which fuels mitochondrial oxidative phosphorylation. However, in the setting of increased tissue lactate accumulation, as shown in the TRACK kidneys (Fig. 1A), we would expect to see a corresponding reduced flux of pyruvate entering the TCA cycle. In addition, our mRNA expression data showed that the HIF1α target, PDK1, a protein that is known to inhibit the TCA cycle [37], is increased in TRACK kidneys (Table 1). Thus, we would predict a diminished level of TCA cycle activity and pathway intermediates in the TRACK kidney tumors. Metabolomics analysis indeed confirmed this hypothesis (Fig. 1B,C). Notably, we found that levels of key intermediates in the TCA cycle, fumarate and α-ketoglutarate, are lower in both the TRACK kidneys and human ccRCC samples (Fig. 1B). Taken together, the metabolomics and mRNA expression data indicate that TRACK kidney tumors exhibit glycolysis and lactate production and reduced flux of pyruvate entering the TCA cycle, which is in association with increased expression of HIF1α target genes that mediate the Warburg effect.

**Fig 2:**
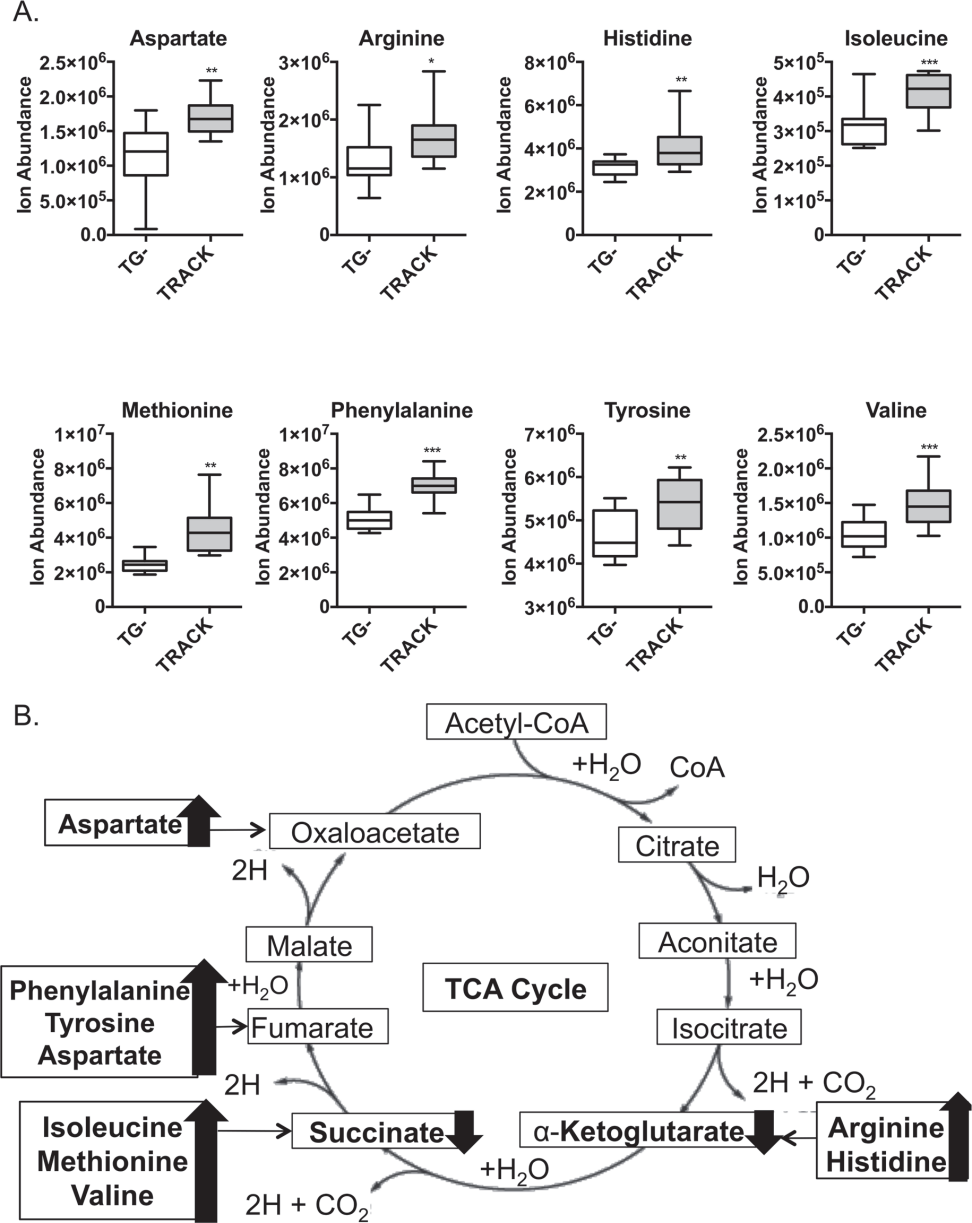
Metabolomic analysis of amino acid levels in TRACK kidneys. A: Levels of amino acids in WT vs. TRACK mouse kidneys (n = 14 and 12). □ = TG^**−**^; ■ = TRACK. B: Schematic overview of changes to amino acid levels and TCA cycle. Error bars indicate the mean ± SD. * indicates a p value <0.05, ** indicates a p value <0.01, and *** indicates a p value <0.001.

Alterations in cancer cell metabolism and energy production are adapted to facilitate the uptake and incorporation of nutrients into biomass, such as amino acids [22]. This is largely because of the high demand of cancer cells for macromolecular building blocks required for cell proliferation. We assessed TRACK kidney derived metabolites and found increased levels of multiple amino acids in the TRACK kidneys as compared to TG^**−**^ kidneys (Fig. 2A,B). Specifically, we detect significant increases in the levels of aspartate, methionine, valine, histidine, tyrosine, isoleucine, phenylalanine, and arginine (Fig. 2A,B). In addition to serving as precursors for protein synthesis, these amino acids can be metabolized to intermediates of the TCA cycle: α-ketoglutarate, succinate, fumarate, and oxaloacetate.

### Analysis of Metabolic Alterations in Human ccRCC Samples

**Fig 3:**
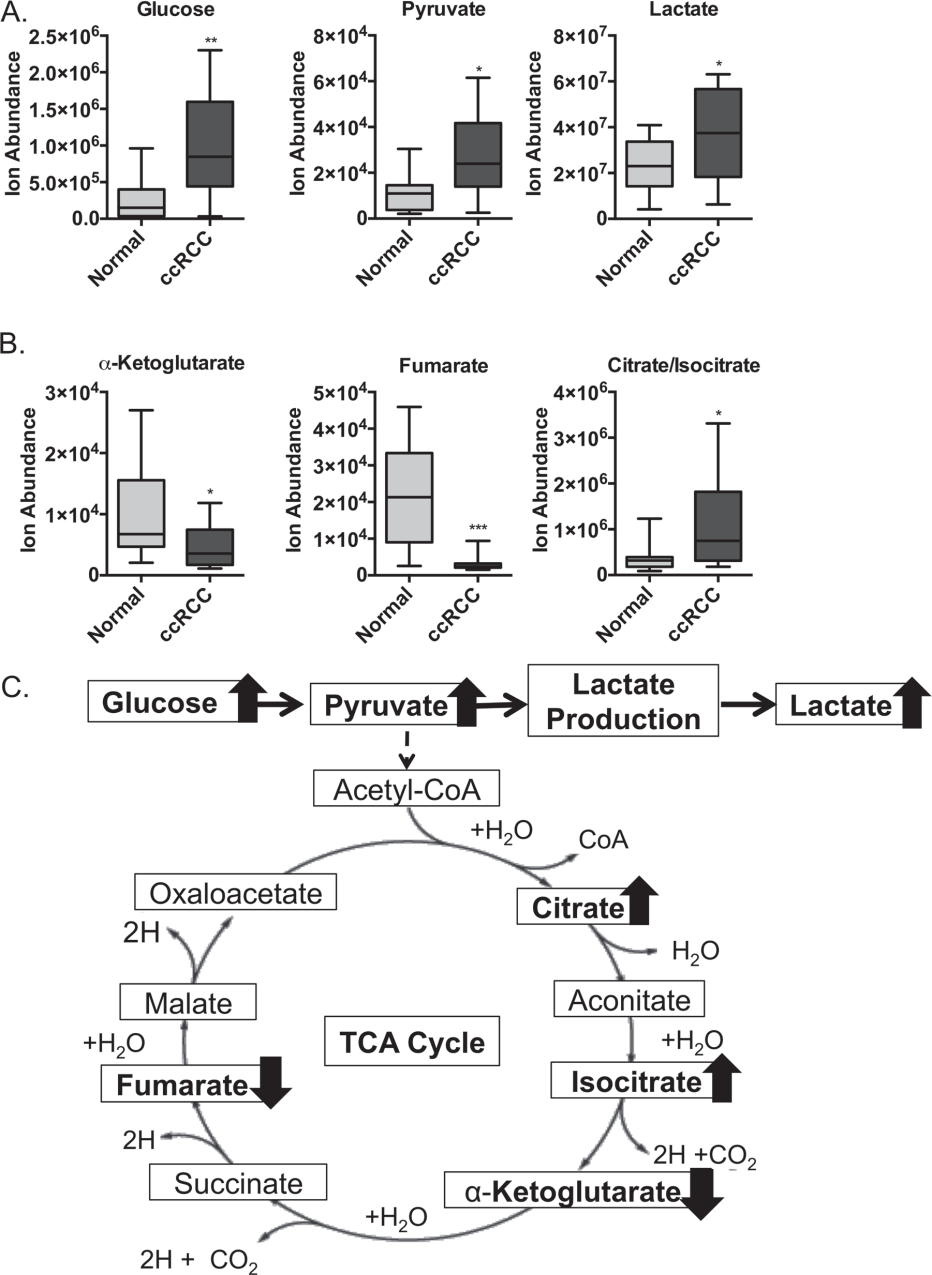
Metabolomic analysis of key intermediates in glycolysis, lactate production, and the TCA cycle in ccRCC samples. A: Comparison of the levels of glycolysis and lactate production intermediates, glucose, pyruvate, and lactate in normal vs. ccRCC samples (n = 12). ■ = Normal; ■ = ccRCC. B: Comparison of the levels of fumarate, α-ketoglutarate, and citrate/isocitrate in normal vs. ccRCC samples (n = 12). ■ = Normal; ■ = ccRCC. C: Schematic overview of changes to glycolysis, lactate production, and the TCA cycle in ccRCC. Error bars indicate the mean ± SD. * indicates a p value <0.05, ** indicates a p value <0.01, and *** indicates a p value <0.001.

Our hypothesis is that the TRACK model is representative of human ccRCC. When we compare the metabolomics analysis of TRACK kidneys to human ccRCC we detect higher levels of pyruvate and lactate in human ccRCCs, similar to what we detect in TRACK kidneys (Fig. 3A). As in the TRACK kidneys, after glucose is taken up in the ccRCC samples it is rapidly metabolized to pyruvate by glycolysis and then converted to lactate (Fig. 3A,C). Additionally, we found a reduction in TCA cycle intermediates in the ccRCC samples, including lower levels of fumarate and α-ketoglutarate (Fig. 3B). Overall, these changes are consistent with the view that both TRACK kidney and human ccRCC cells rely on anaerobic glycolysis instead of on the TCA cycle as a primary bioenergetics source for ATP production.

In the ccRCC samples, we also observe increased levels of the TCA cycle intermediate(s) citrate/isocitrate (these cannot be differentiated by metabolomic analysis) (Fig. 3B), while there were no changes in the levels of these intermediates in the TRACK model (Fig. 1A) Additionally, and in contrast to our findings in the TRACK kidneys, we did not detect changes to the levels of various amino acids in the ccRCC samples (data not shown).

## Discussion

In this study we have demonstrated that TRACK mice exhibit altered tumor metabolism that is highly similar to the changes detected in human ccRCC samples. Our data from RNA sequencing and the Oncomine database indicate that many HIF1α target genes that mediate glucose uptake and glycolysis, suppress the activity of the TCA cycle and mitochondrial respiration, and maintain pH homeostasis during aerobic respiration show increased transcript levels in TRACK mice (Table 1). These results are not surprising, since TRACK mice selectively express a mutant, constitutively active form of HIF1α in the renal proximal tubules where these transcriptome changes are observed [16].

In agreement with these results on mRNA expression, analysis of the metabolome further demonstrates that HIF1α mediates altered metabolism in TRACK kidneys (Figs. 1–2). We found that pyruvate and lactate levels are increased in TRACK kidneys, in accord with an increase in glycolysis and lactate production (Fig. 1A,C). Additionally, reduced levels of α-ketoglutarate and fumarate, metabolites of the TCA cycle, are seen in the TRACK kidneys, suggestive of a reduced flux of pyruvate entering the TCA cycle (Fig. 1B,C). Since the TCA cycle is responsible for generating NADH to fuel mitochondrial oxidative phosphorylation, we also infer that oxidative phosphorylation is reduced in TRACK kidneys. The mRNA expression data corroborate this inference, since we detect higher levels of NDUFA4L2, a known inhibitor of oxidative phosphorylation, in TRACK kidney samples (Table 1). Previous studies have also shown that HIF1α inhibits mitochondrial biogenesis and reduces oxygen consumption in VHL-null renal cell carcinoma [39], which is consistent with our findings. Furthermore, a decrease in SIRT6 expression leads to HIF1α activation that results in increased glucose uptake, with upregulation of glycolysis and diminished mitochondrial respiration [40]. Overall, our data suggest that HIF1α mediates the shift from mitochondrial respiration to glycolysis in TRACK kidneys.

It has long been known that cancer cells acquire and metabolize nutrients in a manner that is conducive to proliferation rather than efficient ATP production [22]. The synthesis of new proteins is required for the enhanced proliferation of cancer cells. In TRACK kidneys we detect increases in the levels of many different amino acids that are required for protein synthesis (Fig. 2A,B). Amino acid uptake has not been well studied in human ccRCC. However, one study has shown that reduced expression of PDK1 by 75–90% in a hypomorphic murine model leads to a reduction in renal absorption of amino acids [41]. This is likely because these mice have decreased expression of various amino acid transporters in the proximal tubule cells [41]. We show that PDK1 is a HIF1α target gene that is highly expressed at the mRNA level in TRACK kidneys (and human ccRCC samples), and that there are increased levels of amino acids in the TRACK kidneys (Fig. 2A,B). Together, these data suggest a link between HIF1α activation and amino acid uptake. Further studies will be needed to determine if HIF1α mediated expression of PDK1 leads to an increase in amino acid uptake in human ccRCC.

Notably, many of the changes to metabolite levels in TRACK kidneys are similar to the changes observed in human ccRCC samples. In human ccRCC samples, we also detect an increase in pyruvate and lactate, and a decrease in TCA cycle metabolites α-ketoglutarate and fumarate (Fig. 3A,B). Thus, in both TRACK kidneys and human ccRCC samples we observe increases in glycolysis and lactate production and a reduced flux of pyruvate entering the TCA cycle (Figs. 1 and 3). These findings suggest that constitutively active HIF1α is sufficient to mediate altered tumor metabolism in TRACK kidneys and likely contributes to the altered metabolism in human ccRCC.

Some of the changes in metabolite levels were dissimilar in the TRACK kidneys and human ccRCC samples. To start, we did not observe an increase in the level(s) of citrate/isocitrate in TRACK kidneys (Fig. 1B) like we did in the human ccRCC samples (Fig. 3B,C). Hypoxia is sufficient to reduce intracellular citrate levels, which in turn induces the anapleurotic process of reductive glutamine metabolism to fuel lipid biosynthesis for rapid cell proliferation [42,43]. In our study, the levels of citrate/isocitrate are trending towards lower in the TRACK kidneys (Fig. 1B), which is in agreement with the data in the literature that high HIF1α expression is sufficient to induce reductive glutamine metabolism [42,44]. However, in ccRCC tumor cells are dependent on reductive glutamine metabolism for lipid biosynthesis [42,44] and thus it is surprising that we detected elevated levels of citrate/isocitrate in ccRCC tumors compared to those in normal kidney tissue (Fig. 3B,C). Additional differences in the TRACK kidneys compared to human ccRCC samples are the changes in amino acids levels. Many amino acids are elevated in TRACK kidney samples (Fig. 2A,B) but there are no changes in these amino acids in ccRCC samples (data not shown). One potential explanation for this difference is that the stages of tumorigenesis in the TRACK kidneys vs. the human ccRCC samples are different. Notably, the human tumor samples are from a more advanced stage of tumorigenesis than the TRACK samples.

Altered tumor metabolism is now a widely accepted hallmark of cancer, and metabolic alterations greatly influence tumor development and progression [19]. We have shown that HIF1α increases transcripts that encode proteins that mediate increased glycolysis, increased lactate production, and decreased mitochondrial respiration in TRACK kidneys. Additionally, our metabolomics analyses further demonstrate that HIF1α mediates altered tumor metabolism in TRACK kidneys, which is highly similar to the changes observed in human ccRCC. It was previously suggested that HIF1α mediates the Warburg effect in human ccRCC, though these earlier data were based on measurements of transcripts alone, without establishing the activities of encoded proteins [45]. Our analyses of the metabolome expand on this prior knowledge and establish the ability of HIF1α to mediate altered tumor metabolism in an informative renal cancer model, the TRACK mouse. Additionally, these data show that the TRACK model is a highly relevant model of metabolic ccRCC that can be utilized for testing novel therapies that target altered tumor metabolism to inhibit ccRCC.

## Supporting Information

S1 Fig Untargeted metabolomic profiling of WT vs. TRACK mouse kidneys, and normal vs. tumor human kidneys. A: Principal component analysis plot of ANP-NEG data showing a three dimensional visualization of similarities and differences between each samples. B: Principal component analysis clustering of ANP-POS data showing a three dimensional visualization of similarities and differences between each sample. C: Unsupervised hierarchical cluster analysis of ANP-NEG data based on the Pearson correlation. D: Unsupervised hierarchical cluster analysis of ANP-POS data based on the Pearson correlation.
(TIF)

S1 Table Overexpressed Genes in Human ccRCC. Data from Oncomine, a cancer microarray database that collects data to compare mRNA levels in human cancer vs. normal tissue. The data were collected from five datasets, each of which collected data from 20–260 patient samples. The rank for each gene is the median rank for that gene across each of the analyses, and the p-value for each gene is its p-value for the median-ranked analysis. The fold change for each gene is listed from each dataset: 1. Hereditary ccRCC, Beroukhim, et al. (2009). 2. Non-Hereditary ccRCC, Beroukhim, et al. (2009). 3. Gumz, et al. (2007). 4. Lenburg, et al. (2003). 5. Yusenko, et al. (2009). The median fold change is the median fold change calculated from the 5 datasets.
(DOCX)
