# Supplementary figures and images for: Analyses of the Transcriptome and Metabolome Demonstrate That HIF1α Mediates Altered Tumor Metabolism in Clear Cell Renal Cell Carcinoma
Source: PLoS One. 2015 Apr 1;10(4):e0120649. doi: 10.1371/journal.pone.0120649 (PMC4382166; doi:10.1371/journal.pone.0120649)

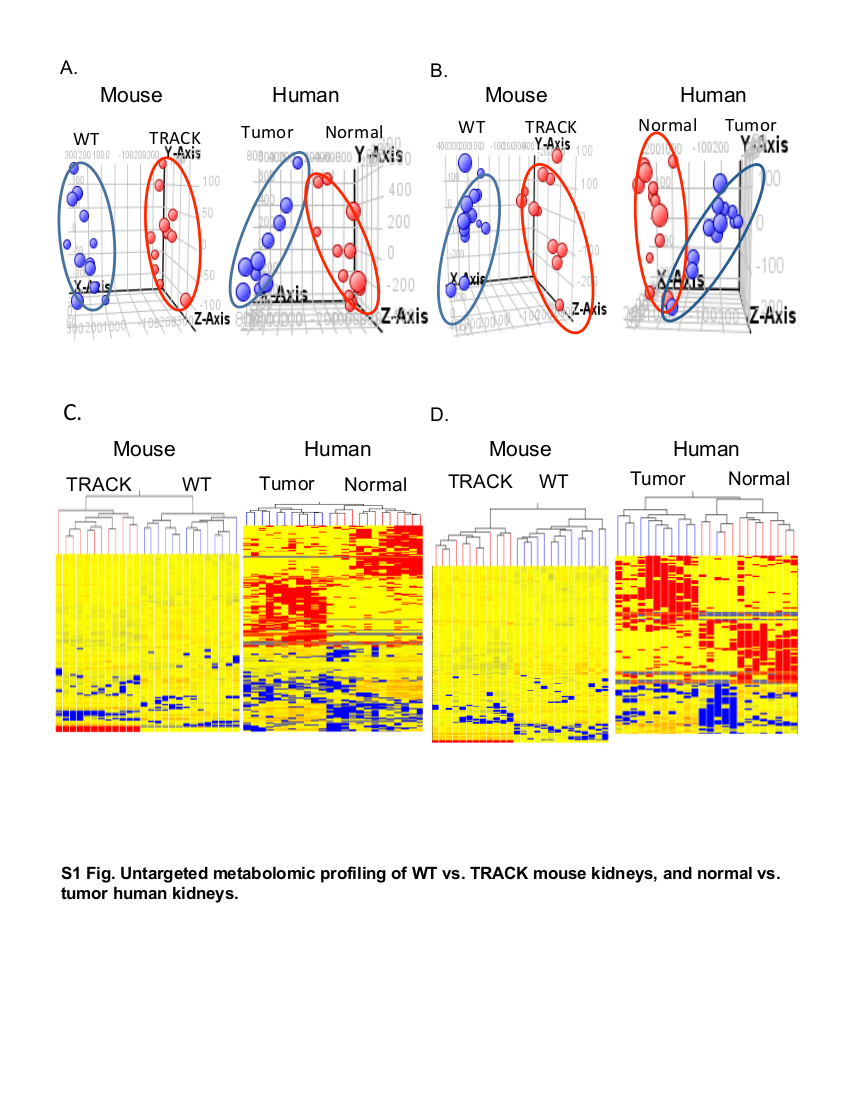

Supplement: S1 Fig — A: Principal component analysis plot of ANP-NEG data showing a three dimensional visualization of similarities and differences between each samples. B: Principal component analysis clustering of ANP-POS data showing a three dimensional visualization of similarities and differences between each sample. C: Unsupervised hierarchical cluster analysis of ANP-NEG data based on the Pearson correlation. D: Unsupervised hierarchical cluster analysis of ANP-POS data based on the Pearson correlation. (TIF) [file pone.0120649.s001.tif]
