# Supplementary material for: Analyses of the Transcriptome and Metabolome Demonstrate That HIF1α Mediates Altered Tumor Metabolism in Clear Cell Renal Cell Carcinoma
Source: PLoS One. 2015 Apr 1;10(4):e0120649. doi: 10.1371/journal.pone.0120649 (PMC4382166; doi:10.1371/journal.pone.0120649)
Supplement: S1 Table — Data from Oncomine, a cancer microarray database that collects data to compare mRNA levels in human cancer vs. normal tissue. The data were collected from five datasets, each of which collected data from 20–260 patient samples. The rank for each gene is the median rank for that gene across each of the analyses, and the p-value for each gene is its p-value for the median-ranked analysis. The fold change for each gene is listed from each dataset: 1. Hereditary ccRCC, Beroukhim, et al. (2009). 2. Non-Hereditary ccRCC, Beroukhim, et al. (2009). 3. Gumz, et al. (2007). 4. Lenburg, et al. (2003). 5. Yusenko, et al. (2009). The median fold change is the median fold change calculated from the 5 datasets. (DOCX) [file pone.0120649.s002.docx]

**Table S1. Overexpressed Genes in Human ccRCC.**

|  | **Median** |  | **Fold Change For Particular Data Set** | | | | | **Median Fold** |
| --- | --- | --- | --- | --- | --- | --- | --- | --- |
| **Gene** | **Rank** | **p-Value** | **1** | **2** | **3** | **4** | **5** | **Change** |
| NDUFA4L2 | 7 | 5.55 x 10 ^-23^ | 75.69 | 50.12 | 53.93 | 23.34 | 86.56 | 52.02 |
| HILPDA | 14 | 3.91 x 10 ^-13^ | 32.38 | 26.75 | 16.47 | 7.58 | 12.89 | 14.68 |
| ENO2 | 14 | 2.82 x 10 ^-11^ | 10.28 | 12.47 | 25.79 | 6.63 | 19.97 | 16.22 |
| EGLN3 | 16 | 9.5 x 10 ^-19^ | 36.18 | 21.97 | 12.10 | 5.01 | 12.16 | 12.13 |
| IGFBP3 | 21 | 1.73 x 10 ^-14^ | 14.70 | 8.41 | 12.33 | 10.96 | 10.15 | 10.56 |
| SPAG4 | 25 | 1.23 x 10 ^-10^ | 13.93 | 12.23 | 11.13 | 3.99 | 26.72 | 11.68 |
| AHNAK2 | 28 | 2.83 x 10 ^-17^ | 9.86 | 15.08 | 15.04 | 5.85 | 18.20 | 15.06 |
| TMCC1 | 30 | 3.79 x 10 ^-7^ | 9.69 | 8.36 | 6.05 | 4.62 | 13.47 | 7.20 |
| RNASET2 | 36 | 2.19 x 10 ^-16^ | 8.96 | 6.96 | 10.96 | 7.33 | 8.56 | 7.94 |
| CAV1 | 38 | 1.93 x 10 ^-13^ | 8.39 | 7.08 | 8.17 | 9.38 | 5.03 | 7.62 |
| SLC16A3 | 41 | 2.54 x 10 ^-13^ | 8.46 | 6.66 | 7.13 | 5.87 | 15.70 | 6.89 |
| LCP2 | 46 | 6.84 x 10 ^-10^ | 5.72 | 4.57 | 3.78 | 1.33 | 6.56 | 4.18 |
| CA9 | 47 | 6.92 x 10 ^-16^ | 27.90 | 17.97 | 14.48 | 9.18 | 37.68 | 16.23 |
| NETO2 | 48 | 7.84 x 10^-10^ | 18.10 | 15.43 | 25.58 | 6.04 | 4.73 | 10.73 |
| IFNGR2 | 49 | 8.25 x 10 ^-10^ | 2.83 | 2.68 | 2.61 | 1.74 | 2.47 | 2.54 |
| ABCG1 | 57 | 3.12 x 10 ^-15^ | 4.92 | 5.97 | 3.45 | 2.26 | 4.90 | 4.18 |
| UBE2L6 | 57 | 1.37 x 10 ^-12^ | 2.67 | 2.33 | 1.98 | 2.06 | 3.09 | 2.19 |
| DIAPH2 | 59 | 1.59 x 10 ^-10^ | 3.25 | 3.64 | 2.73 | 1.88 | 3.33 | 3.03 |
| ALDOA | 59 | 1.50 x 10 ^-9^ | 2.42 | 2.34 | 2.32 | 2.63 | 3.26 | 2.49 |
| VEGFA | 60 | 6.13 x 10 ^-15^ | 8.56 | 6.67 | 3.81 | 3.15 | 4.75 | 4.28 |
| TAPBP | 63 | 7.81 x 10 ^-15^ | 2.75 | 2.53 | 2.82 | 2.88 | 4.26 | 2.85 |
| ALDOC | 63 | 1.82 x 10 ^-12^ | 7.65 | 6.46 | 2.81 | 2.46 | 5.52 | 4.16 |
| PFKP | 64 | 2.13 x 10 ^-6^ | 7.14 | 5.72 | 8.39 | 5.98 | 10.13 | 7.18 |
| BTN3A3 | 65 | 2.18 x 10 ^-6^ | 4.72 | 3.13 | 5.15 | 3.70 | 4.33 | 4.02 |
| LDHA | 69 | 1.17 x 10 ^-14^ | 2.86 | 2.68 | 3.09 | 2.32 | 3.46 | 2.88 |
| PDK1 | 70 | 2.88 x 10 ^-9^ | 2.95 | 2.11 | 8.18 | 6.98 | 6.98 | 6.98 |
| BHLHE41 | 79 | 2.89 x 10^-6^ | 18.41 | 20.32 | 11.57 | 6.08 | 28.99 | 15.94 |
| TRIB3 | 81 | 3.52 x 10 ^-14^ | 4.23 | 5.90 | 3.68 | 2.66 | 7.20 | 4.79 |
| SCARB1 | 83 | 4.72 x 10 ^-14^ | 5.04 | 6.48 | 5.93 | 3.79 | 8.93 | 6.21 |
| ANXA4 | 83 | 6.46 x 10 ^-12^ | 2.89 | 2.74 | 3.56 | 3.01 | 4.76 | 3.28 |
